# An *In Vitro* Study on the Effect of Free Amino Acids Alone or in Combination with Nisin on Biofilms as well as on Planktonic Bacteria of *Streptococcus mutans*

**DOI:** 10.1371/journal.pone.0099513

**Published:** 2014-06-17

**Authors:** Zhongchun Tong, Luodan Zhang, Junqi Ling, Yutao Jian, Lijia Huang, Dongmei Deng

**Affiliations:** 1 Department of Operative Dentistry and Endodontics, Guanghua School of Stomatology, Sun Yat-sen University, Guangzhou, Guangdong, China; 2 Guangdong Provincial Key Laboratory of Stomatology, Sun Yat-sen University, Guangzhou, Guangdong, China; 3 Department of Cariology Endodontology Pedodontology, Academic Centre for Dentistry Amsterdam (ACTA), Universiteit van Amsterdam and Vrije Universiteit, Amsterdam, The Netherlands; University of Oklahoma Health Sciences Center, United States of America

## Abstract

Free D-amino acids (D-AAs) are one of the most striking features of the peptidoglycan composition in bacteria and play a key role in regulating and disassembling bacterial biofilms. Previous studies have indicated that the antimicrobial peptide nisin can inhibit the growth of the cariogenic bacteria *Streptococcus mutans*. The present study investigated the effect of free amino acids either alone or in combination with nisin on biofilm and on planktonic *S. mutans* bacteria. The results of the MIC and MBC analyses showed that D-cysteine (Cys), D- or L-aspartic acid (Asp), and D- or L-glutamic acid (Glu) significantly improve the antibacterial activity of nisin against *S. mutans* and that the mixture of D-Cys, D-Asp, and D-Glu (3D-AAs) and the mixture of L-Cys, L-Asp, and L-Glu (3L-AAs) at a concentration of 40 mM can prevent *S. mutans* growth. Crystal violet staining showed that the D- or L-enantiomers of Cys, Asp, and Glu at a concentration of 40 mM can inhibit the formation of *S. mutans* biofilms, and their mixture generated a stronger inhibition than the components alone. Furthermore, the mixture of the three D-AAs or L-AAs may improve the antibacterial activity of nisin against *S. mutans* biofilms. This study underscores the potential of free amino acids for the enhancement of the antibacterial activity of nisin and the inhibition of the cariogenic bacteria *S. mutans* and biofilms.

## Introduction

Biofilms are complex microbial communities. The microorganisms in biofilms are generally encapsulated by extracellular polymeric substances and tightly adhered to different surfaces [1], [2]. Because biofilms are difficult to eradicate and are resistant to antimicrobial agents, biofilms have caused a range of problems and profoundly affect human health [3]. The inhibition of biofilms remains one of the crucial problems that current microbiologists aim to overcome. Recently, some researchers have investigated biofilm detachment, and some biofilm dispersants, such as D-amino acids (D-AA), dispersin B, bacteriophage, and polyamine, have been found to disperse biofilms [4], [5], [6]. Brandenburg et al. reported that tryptophan inhibits *Pseudomonas aeruginosa* biofilm formation on tissue culture plates and further inhibits the growth of existing biofilms [7]. Kolodkin-Gal et al. reported that D-leucine, D-methionine, D-tyrosine, and D-tryptophan prevent *Bacillus subtilis* biofilm formation and can also break down existing biofilms [5].

Dental plaque, which consists of a matrix of polysaccharides, proteins, and microbial cells, is a typical microbial biofilm. *Streptococcus mutans* is a major cariogenic bacterium that plays a decisive role in the development of dental plaque [8], [9]. *S. mutans* can utilize extracellular sucrose to produce exopolysaccharide glucan, which is a key component of the dental plaque biofilm and promotes the further adherence and accumulation of additional cariogenic microorganisms on the surface of teeth [10], [11], [12], [13]. Thus, the inhibition of *S. mutans* biofilms favors the prevention of dental plaque formation and thereby reduces the incidence of dental caries.

D-AAs are one of the most striking features of a peptidoglycan composition [14], [15]. D-AAs were recently found to play very important roles in regulating and disassembling bacterial biofilms and may provide a general strategy for the prevention of biofilm [5], [14]. However, to date, little information is available on the effects of exogenous D-AAs on the cariogenic bacterium *S. mutans* and on biofilms. In our previous studies, the antimicrobial peptide nisin showed bactericidal activity against *S. mutans* by the formation of pores on the surface of cell membranes and damaging the cell walls [16], [17]. The present study attempted to investigate whether 18 free D-amino acids improve the antibacterial activity of three common antimicrobials, namely nisin, chlorhexidine, and penicillin, against *S. mutans* and further evaluated the effects of these available D-amino acids either alone or in conjunction with nisin on *S. mutans* biofilms.

## Materials and Methods

### Bacterial Strains and Growth Conditions

*Streptococcus mutans* UA159, which was obtained from freezer stocks, was streaked on brain-heart infusion (BHI, Becton Dickinson, Sparks, MD, USA) agar plates and placed in an anaerobic jar containing 85% N_2_, 10% H_2_, and 5% CO_2_ at 37°C for 24 h. A single colony was inoculated into 5 mL of BHI broth and incubated to the exponential phase of growth. The optical density (OD) at 600 nm of the *S. mutans* culture was adjusted to 0.55 (Infinite M200, Tecan Group Ltd., Austria), which corresponds to a bacterial concentration of approximately 10^9^ CFU/mL.

### Amino Acid and Nisin Preparation

D-methionine, D-tryptophan, D-leucine, D- or L-cysteine, D- or L-aspartic acid, D- or L-glutamic acid, D-alanine, D-threonine, D-valine, D-isoleucine, D-glutamine, D-phenylalanine, D-histidine, D-proline, D-lysine, D-arginine, and glycine (Sigma-Aldrich, St. Louis, MO, USA) were all prepared at a concentration of 40 mM, and D-tyrosine was prepared at a concentration of 0.8 mM due to its low solubility. Nisin powder (2.5% purity, Sigma-Aldrich, St. Louis, MO, USA) was dissolved in a solution at pH 2 to obtain a stock solution with a concentration of 1 g/L.

### MIC and MBC

The MICs and MBCs of nisin in combination with D-AAs or L-amino acids (L-AAs) against *S. mutans* were determined using the Clinical and Laboratory Standards Institute (CLSI) standards. Briefly, a total volume of 200 µL, including D- or L-amino acids, different concentrations of nisin, the *S. mutans* culture (at a final concentration of 5×10^5^ CFU/mL), and the MHB medium, was added to a 96-well microplate (Costar, Corning Inc., Corning, NY, USA). The working concentrations of nisin were 50, 37.5, 25, 20, 15, 10, 5, 2.5, and 1.25 mg/L, and the working concentration of the amino acids was 10 mM. The microplate was incubated at 37°C for 48 h. The MIC is the lowest concentration of an antimicrobial that will inhibit the visible growth of *S. mutans*. For the MBC calculation, after the content of each well was thoroughly mixed, 10 µL was removed from the well that defined the MIC and the wells with concentrations above the MIC, and these aliquots were plated onto BHI agar plates. The MBC was defined as the lowest concentration of an antibiotic that killed at least 99.9% of the bacteria in a given time. Furthermore, the same procedure was used to evaluate the effect of the mixture of D-Cys, D-Asp, and D-Glu (3D-AAs) and the mixture of L-Cys, L-Asp, and L-Glu (3L-AAs) at a concentration of 10 mM on the antibacterial activity of nisin and the MIC and MBC of penicillin or chlorhexidine in combination with D- or L-amino acids. Each test was repeated five times on different days.

### Time-kill Study with Planktonic Bacteria

The *S. mutans* culture in the exponential phase was adjusted with fresh BHI broth to obtain a concentration of approximately 5×10^5^ cells/mL and subsequently challenged with (1) the mixture of 3D-AAs at their respective concentration of 40 mM, (2) the mixture of 3L-AAs at their respective concentration of 40 mM, (3) 3D-AAs at their respective concentration of 40 mM (pH 7), (4) 3L-AAs at their respective concentration of 40 mM (pH 7), (5) 3D-AAs at their respective concentration of 10 mM, (6) 3L-AAs at their respective concentration of 10 mM, (7) 3D-AAs at their respective concentration of 5 mM, (8) 3L-AAs at their respective concentration of 5 mM, and (9) control (without AAs) at 37°C under anaerobic conditions. The bacterial survival was counted by plate counts of the viable cells at 4, 8, 12, and 24 h. All of the determinations were repeatedly performed three times on different days.

### Crystal Violet (CV) Staining Assay

The CV staining assay was performed as described by Zhou *et al*. [18]. D-Cys, D-Asp, and D-Glu or their L-amino acids at concentrations of 40 mM, 20 mM, or 10 mM were prepared with BHIS (BHI broth with 1% sucrose). The exponential-phase *S. mutans* culture and BHIS broth, including amino acids at a ratio of 1∶100, were added into a 96-well microplate (semi-quantitative analysis) and a 24-well microplate (visualization). After 24 h of incubation, the biofilms on the bottom of the two microplates were stained with 0.1% crystal violet for 15 min and then washed with sterile distilled water. The bound dye was released by the addition of 33% acetic acid and examined using a microplate reader at 575 nm. Before the measurement, the test elution liquid of crystal violet was diluted 1∶4 with distilled water because the original readings were beyond the range of the microplate reader. The same procedure was used to evaluate the effect of the 3D-AAs and 3L-AAs at their respective concentrations of 40 mM, 20 mM, 10 mM, or 5 mM on *S. mutans* biofilm formation. The OD of the *S. mutans* biofilm at 575 nm was assayed after 2, 12, and 24 h of incubation. Furthermore, to eliminate the effect of the acids of amino acids on biofilm formation, the 3D- or 3L-AAs were adjusted to pH 7 with sodium hydroxide solution, and the *S. mutans* biofilms were evaluated through CV staining.

The same CV staining was used to evaluate the effect of 3D- or 3L-AAs on existing biofilms. Briefly, a *S. mutans* culture was incubated in BHIS broth and cultured for 24 h. Subsequently, the 24-h-old biofilms were exposed to 3D-AAs or 3L-AAs at their respective concentration of 40 mM for 24 h and then were assayed by CV staining. All 24-well plates subjected to CV staining were analyzed using a digital camera. Each test was repeated five times in three independent experiments.

### Confocal Laser Scanning Microscopy

After treatment with amino acids and nisin, the *S. mutans* biofilms were visualized by confocal laser scanning microscopy (CLSM). A complete tooth was donated by a healthy volunteer whose mandibular third molar was extracted due to pericoronitis, and used in this study by Institution Review Board approval of Guanghua School of Stomatology, Sun Yat-sen University, China. The tooth was transversely cut into 1-mm-thick pieces using a silicon carbide disc (Isomet; 10.2 cm×0.3 mm, Buehler Ltd, Lake Bluff, America). The tooth pieces were soaked in 75% alcohol for 1 h, dried in a super clean bench, soaked in 10 mL of sterile saliva for 2 h (Sterile saliva collected from the oral cavity of a healthy volunteer, and prepared according to Zhou et al. [19]), and placed in a 12-well plate. For biofilm formation, *S. mutans* culture and BHIS at a 1∶100 ratio were added to the 12-well plate, and the mixture was incubated at 37°C for 24 h. The *S. mutans* biofilms on the surfaces of the tooth pieces were exposed to the following solutions at 37°C for 1 h: (A) BHIS (negative control), (B) 125 mg/L nisin, (C) 40 mM 3L-AAs and 125 mg/L nisin, and (D) 40 mM 3D-AAs and 125 mg/L nisin. The four groups of biofilms were stained at room temperature in the dark for 15 min using 6 µM SYTO 9 stain and 30 µM PI (LIVE/DEAD BacLight bacterial viability kit L13152; Molecular Probes, Invitrogen). The images of the stained specimens were captured using a Carl Zeiss LSM 780 instrument and analyzed using the ZEN software (Zen 2012 light edition, Carl Zeiss MicroImaging, Inc., Thornwood, NY, USA). SYTO 9 and PI were excited at 488 nm and 543 nm, respectively. The three-dimensional *S. mutans* biofilms exposed to the drugs were scanned along the Z axis as a subset of 20 layers from bottom to top. The middle layers (tenth layer) from five random three-dimensional images in each group were used to analyze the ratio of dead to live bacteria by fluorescence intensity.

### Statistical Analysis

The statistical analyses were performed using the SPSS 18.0 software. After Bonferroni-correction, Student’s *t*-test was used to evaluate any significant differences between (1) the *S. mutans* survival rates obtained after exposure to different concentrations of 3D-AAs or 3L-AAs and the control at the same time point, and a value of *p*<0.00625 was considered significant; (2) the OD_575_ values of *S. mutans* biofilms obtained in the presence of challenges with different concentrations of Cys, Asp, or Glu and with no amino acids (control), and a value of *p*<0.00833 was considered significant; (3) the OD_575_ values of *S. mutans* biofilms obtained in the presence of challenges with different concentration of 3D-AAs or 3L-AAs and with no amino acids (control) at the same time point, and a value of *p*<0.00625 was considered significant. For all the comparison of D- and L-enantiomers of the same amino acids, and a value of *p*<0.05 was considered significant. The ratios of the fluorescence intensity of dead/live cells were compared by Kruskal-Wallis H and Nemenyi nonparametric tests. A *p* value of less than 0.05 was considered significant.

## Results

### Antibacterial Activity

The MIC and MBC values of nisin against *S. mutans* were 20 and 37.5 mg/L, respectively. As free amino acids, the addition of D-Cys, D-Asp, and D-Glu can significantly improve the antibacterial activity of nisin, and the addition of D-Val, D-Phe, D-Leu, D-Ile, D-Thr, D-Pro, D-Tyr, and D-Ser showed slight improvement. In contrast, the addition of D-Ala, D-Lys, D-Met, D-Trp, D-His, and D-Gly resulted in no improvement, and even the addition of D-Arg decreased the antibacterial activity of nisin. The combination of the three effective amino acid D-Cys, D-Asp, and D-Glu (3D-AAs) displayed inhibition for *S. mutans* even in the absence of nisin, and the same inhibition was observed after treatment with 3L-AAs. Furthermore, to exclude the effect of acid of amino acids on antibacterial activity, 3D-AAs was adjusted to pH 7, and still showed improvement to the antibacterial activity of nisin, but 3L-AAs at pH 7 did not (Table 1). All D-AAs at 10 mM did not change the antibacterial activity of penicillin or chlorhexidine.

**Table 1:** MIC and MBC of nisin in combination with D-amino acids for *S. mutans*.

AA(10 mM)	Nisin (mg/L)	
	MIC	MBC
PBS	20(1.58)	37.5(3.95)
D-Asp*	10(1.58)	20(1.58)
L-Asp	15(2.24)	20(1.58)
D-Ala	19(1.00)	40(2.50)
D-Val	20(1.58)	19(1.87)
D-Phe	15(2.24)	20(1.58)
D-Leu	20(0)	26.5(2.92)
D-Ile	20(1.58)	25(0)
D-Lys	35(2.50)	37.5(3.95)
D-Thr	20(1.58)	26.5(2.92)
D-Met	21(1.00)	37.5(3.95)
D-Trp	20(2.24)	37.5(0)
D-His	20(0)	37.5(3.95)
D-Arg	50(0)	50(0)
D-Cys*	10(1.58)	10(1.58)
L-Cys	19(1.00)	37.5(0)
D-Glu*	10(1.58)	20(1.58)
L-Glu*	11(1.00)	15(2.24)
D-Pro	15(1.58)	24(1.00)
D-Tyr^Δ^	14(1.00)	26.5(2.92)
D-Ser	16(1.00)	16(1.87)
D-Gly	20(0)	37.5(3.95)
3D-AA*	0	4.5(0.50)
3L-AA*	0	5.5(1.22)
3D-AA (pH 7)*	3.75(0.79)	12(2.00)
3L-AA (pH 7)	20(1.58)	37.5(0)

The asterisk indicates that the addition of free amino acids at 10 mM generated significant effect on antibacterial activities of nisin, and decreased the MICs and MBCs of nisin against *S. mutans*. D-tyrosine was prepared at a concentration of 0.8 mM due to its low solubility. The combinations of D-Asp, D-Cys, and D-Glu (3D-AA) or the combinations of L-Asp, L-Cys, and L-Glu (3L-AA) were more effective than the amino acids alone in the improvement of the antibacterial activities of nisin, and even the mixture of the three amino acids at their respective concentration of 10 mM inhibited *S. mutans* in the absence of nisin (MIC of nisin = 0). Each test was repeated five times on different days, and means and standard errors were given.

### Effect of 3D-AA and 3L-AA on *S. mutans* Growth

**Figure 1:**
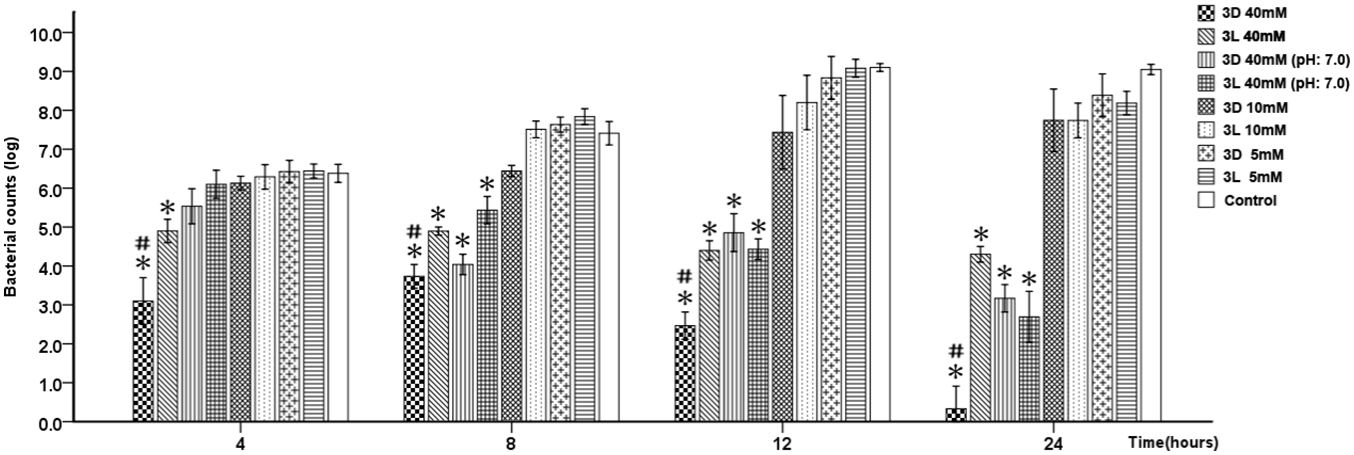
Effect of 3D-AAs and 3L-AAs on *S. mutans* growth. All of the determinations were repeatedly performed three times on different days. The y-axis represents the survival count of bacteria (mean ± SD), and the x-axis represents the time after the challenge with amino acids. “*” indicates a significant difference in the *S. mutans* survival count between the amino acid challenge groups and the control, and a value of *p*<0.00625 was considered significant; “#” indicates a significant difference in the *S. mutans* survival count between the 3D-AA and 3L-AA challenge groups, and a value of *p*<0.05 was considered to be significant.

In the time-kill assay, the 3D-AAs at their respective concentration of 40 mM was the most effective treatment for the inhibition of *S. mutans* of all of the experimental groups, and the *S. mutans* survival rates showed a significant reduction from 4 h to 24 h of challenge, but 3L-AAs exhibited no significant effect after 4 h of challenge. Both treatments showed significant differences at the same time points (*p*<0.05). The adjustment of 3D-AAs and 3L-AAs pH 7 resulted in decreases in the *S. mutans* survival rate after 8, 12, and 24 h of challenge. Significant differences were found between the different experimental groups at 40 mM and the control at the same time point, with the exception of 3D-AAs and 3L-AAs pH 7 at 4 h after challenge (*p*<0.00625). Furthermore, both 3D-AAs and 3L-AAs at concentrations of 10 mM or 5 mM did not significantly decrease the *S. mutans* survival rates (Fig. 1).

### Effect of Cys, Asp, and Glu Alone or in Combination on *S. mutans* Biofilm

**Figure 2:**
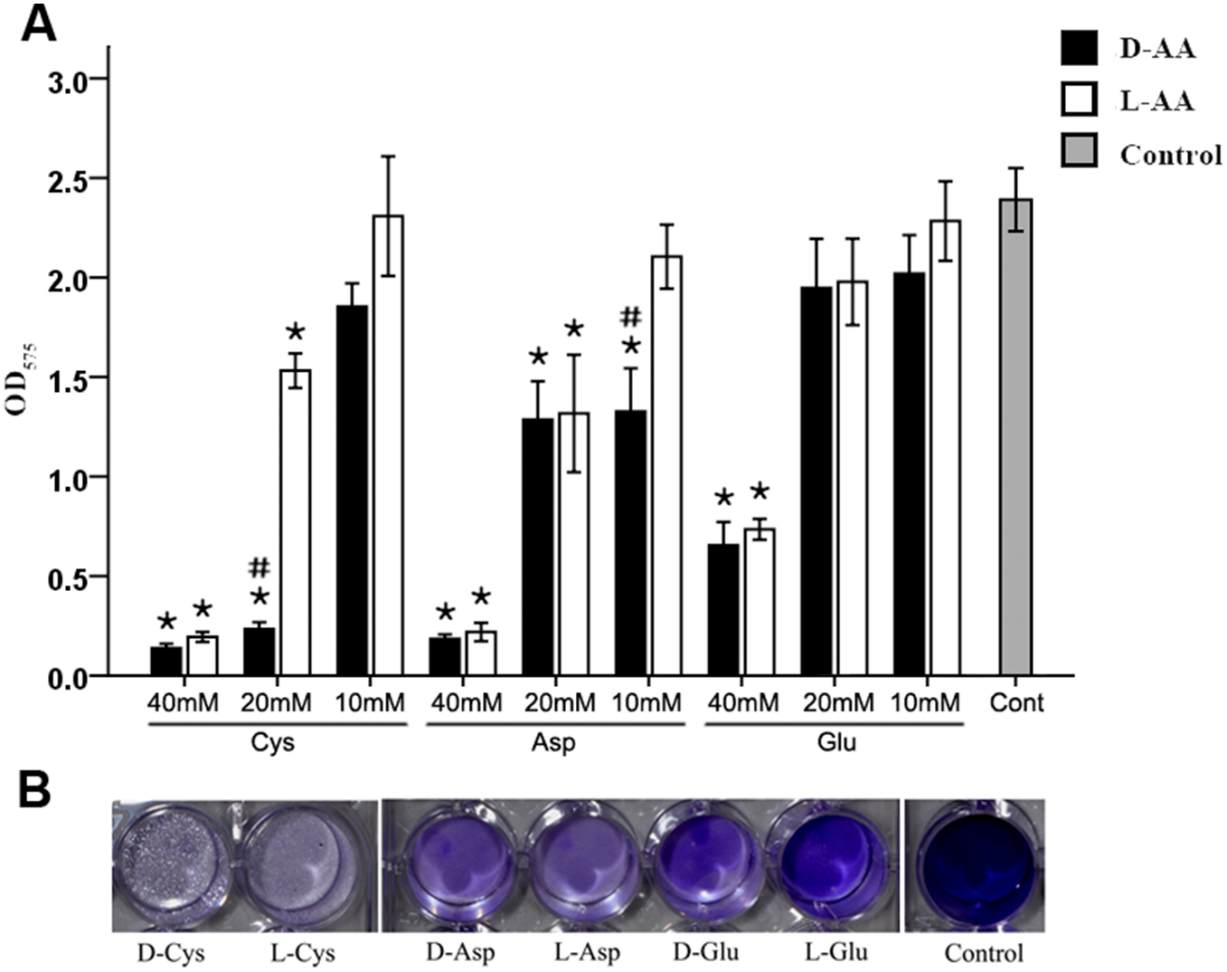
Effect of different concentrations of Cys, Asp, and Glu on *S. mutans* biofilm formation. (A) The quantification of *S. mutans* biofilm formation was evaluated by crystal violet staining after 24 h of challenge with Cys, Asp, and Glu at 40 mM, 20 mM, and 10 mM. The results show the average of three independent experiments repeated five times, and mean and standard deviation were shown. “*” indicates a significant difference in the OD value of *S. mutans* biofilm between the amino acid challenge groups and the control, and a value of *p*<0.00833 was considered significant; “#” indicates a significant difference in the OD value of the *S. mutans* biofilm between the groups challenged with the D- and L-enantiomers of the same amino acid, and a value of *p*<0.05 was considered significant. (B) *S. mutans* culture and BHIS at a 1∶100 ratio were added into 24-well plates and challenged with D- or L-Cys, -Asp, and -Glu at 40 mM for 24 h. The *S. mutans* biofilm formation was visualized by crystal violet staining.

In the biofilm assay, both D- and L-enantiomers of Cys, Asp, and Glu at their respective concentration of 40 mM could significantly inhibit *S. mutans* biofilm formation, but the other amino acids did not. The antibiofilm activity of Cys and Asp were superior to that of Glu. At a concentration of 20 mM, Glu did not significantly prevent biofilm formation, but Cys and Asp were effective. At 10 mM, Cys, Asp, and Glu, with the exception of D-Asp, hardly prevented biofilm formation (Fig. 2).

**Figure 3:**
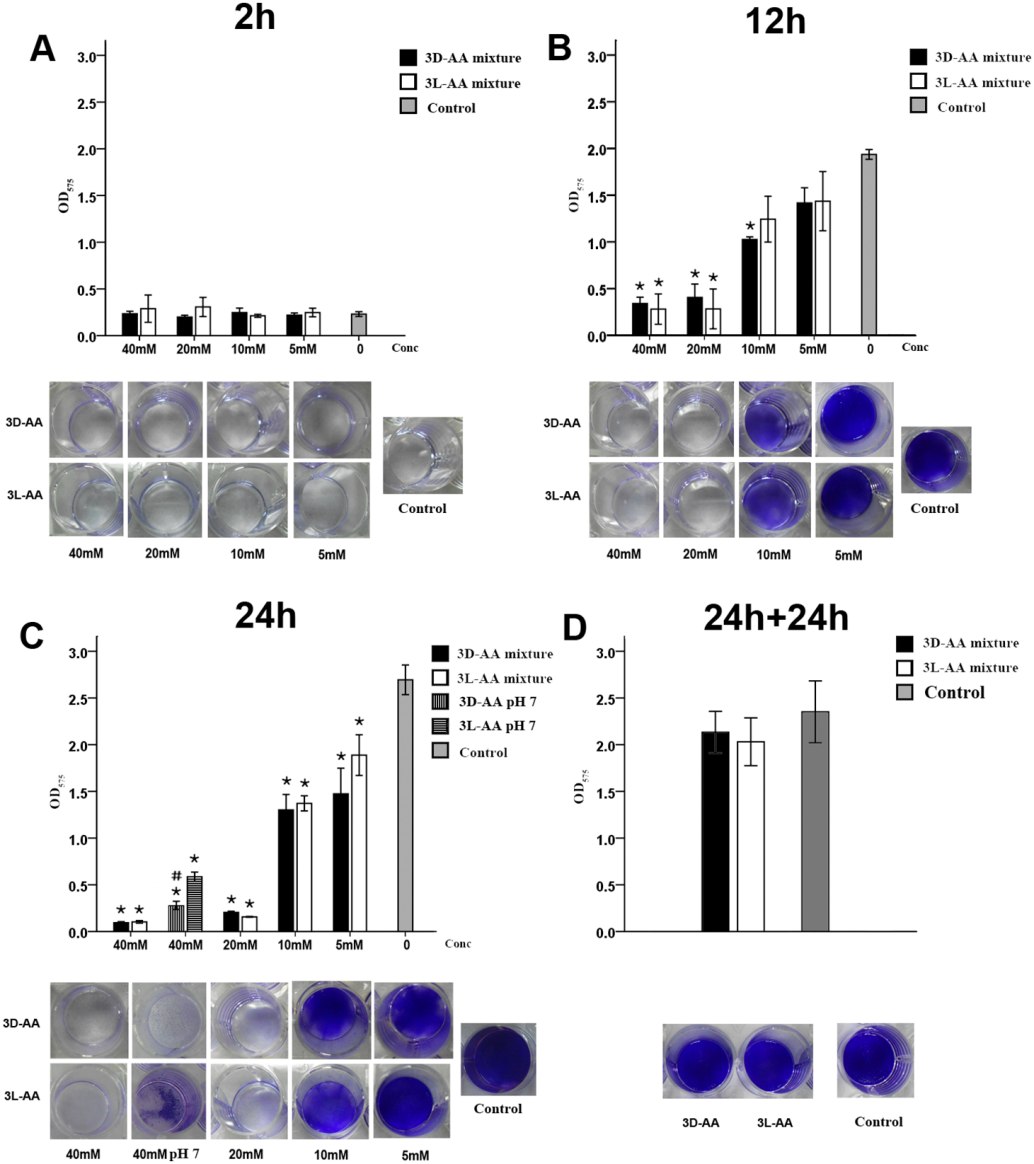
Effect of the mixture of D-Cys, D-Asp, and D-Glu at 40 mM (3D-AA) and the mixture of L-Cys, L-Asp, and L-Glu at 40 mM (3L-AA) on *S. mutans* biofilms. *S. mutans* culture and BHIS at a ratio of 1∶100 were added to 96-well plates (semi-quantitative analysis) and a 24-well microplate (visualization) and were challenged with different concentrations of 3D-AAs or 3L-AAs for 2 h (A), 12 h (B), and 24 h (C), and the *S. mutans* biofilm formation was quantitatively evaluated by crystal violet staining. (D) Effect of 3D-AAs or 3L-AAs at 40 mM on existing *S. mutans* biofilms. *S. mutans* culture and BHIS at a ratio of 1∶100 were cultured for 24 h. The existing biofilm was exposed to 3D- or 3L-AAs for 24 h and quantified by CV staining. The results are an average of three independent experiments repeated five times, and mean and standard deviation were shown. “*” indicates a significant difference in the OD value of *S. mutans* biofilm between the groups challenged with 3D- or 3L-AAs and the control group, and a value of *p*<0.00625 was considered significant; “#” indicates a significant difference in the OD value of the *S. mutans* biofilm between the groups challenged with 3D-AA and 3L-AA at pH 7, and a value of *p*<0.05 was considered significant.

The mixture of Cys, Asp, and Glu showed a greater antibiofilm effect against *S. mutans* than the components alone. After 2 h of exposure to 3D- or 3L-AAs, the OD of *S. mutans* biofilms was almost the same as that of the control (Fig. 3A). After 12 h of treatment, the 3D- or 3L-AAs at their respective concentration of 40 mM or 20 mM and 3D-AAs at their respective concentration of 10 mM inhibited *S. mutans* biofilms, and the experimental and control groups showed significant differences (*p*<0.00625) (Fig. 3B). After 24 h of treatment, both 3D- and 3L-AAs at their respective concentration of 40 mM, 20 mM, 10 mM, or 5 mM showed different levels of inhibition compared to the control (*p*<0.00625). Furthermore, 40 mM 3D- and 3L-AAs at pH 7 also showed an inhibitory effect on biofilm, but the level of inhibition obtained at pH 7 was slightly lower than that obtained with the original pH (Fig. 3C). The subsequent analysis showed that 3D- and 3L-AAs at a concentration of 40 mM are incapable of breaking down existing biofilms (Fig. 3D).

### Assay of the Antibiofilm Activity of Nisin in the Absence and Presence of 3D-AAs and 3L-AAs

**Figure 4:**
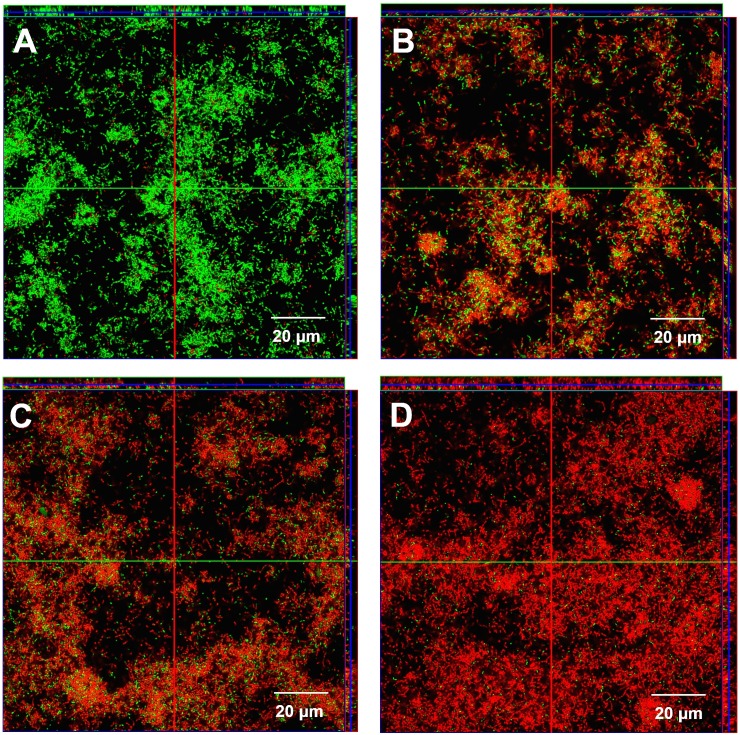
Confocal laser scanning microscopic images of existing *S. mutans* biofilm (24-h-old) on the surfaces of tooth pieces challenged for 1 h. , with (A) BHIS (negative control), (B) 125 mg/L nisin, (C) 40 mM 3L-AAs and 125 mg/L nisin, and (D) 40 mM 3D-AAs and 125 mg/L nisin. The cells with intact membranes are stained green, whereas the cells with damaged cell membranes are stained red. The four groups of three-dimensional *S. mutans* biofilms were captured using a 60×oil lens of a Carl Zeiss CLSM instrument and analyzed using the ZEN software, and the films were scanned along the Z axis as a subset of 20 layers from bottom to top.

**Figure 5:**
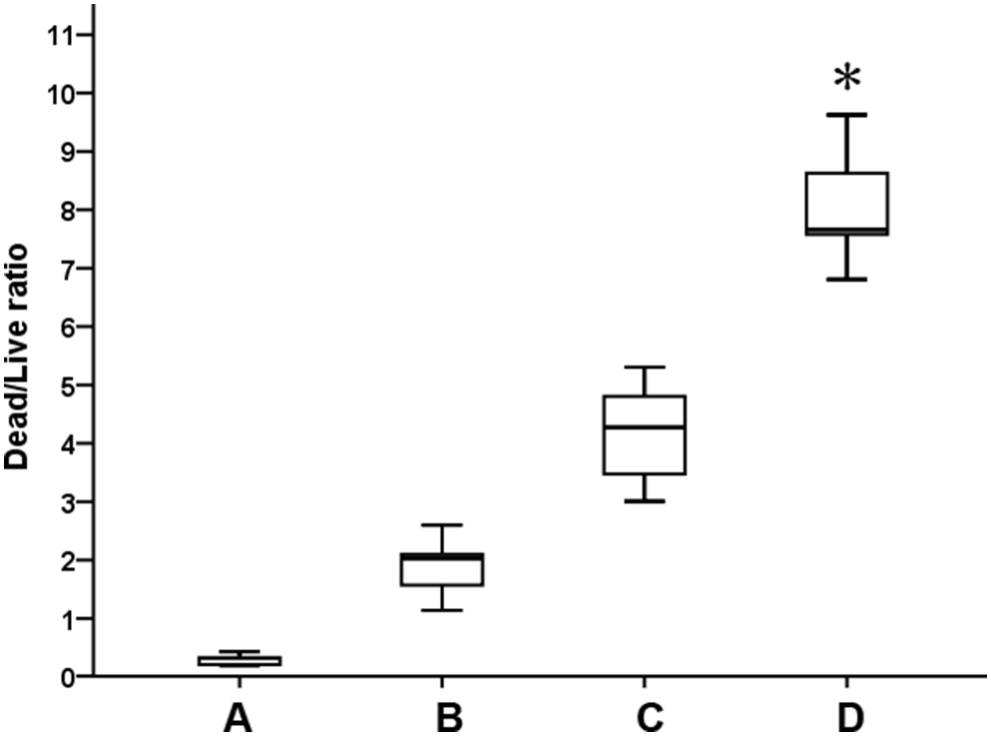
Boxplot depicting the ratios in the fluorescence intensity values of dead to live bacteria (Median, Q_L_/Q_U_). The existing *S. mutans* biofilm (24-h-old) on the surfaces of tooth pieces were challenged for 1 h with (A) BHIS (negative control), (B) 125 mg/L nisin, (C) 40 mM 3L-AAs and 125 mg/L nisin, and (D) 40 mM 3D-AAs and 125 mg/L nisin. Five random three-dimensional images from each group were used to analyze the ratio in the fluorescence intensity of dead to live bacteria. “*” indicates a significant difference in the fluorescence intensity ratios of dead to live bacteria between the C and D treatment groups (*p*<0.05).

Both 3D-AAs and 3L-AAs were found to contribute to the bactericidal activities of nisin against *S. mutans* biofilms, even though they did not generate a significant effect in biofilm breakdown, as determined through a CV staining assay. In the three-dimensional view, more dead cells were found in the nisin treatment group compared with the control (Figs. 4A and 4B). The bactericidal activities of nisin against *S. mutans* biofilms were significantly improved by 3L- AAs and 3D-AAs at their respective concentration of 40 mM (Fig. 4C and 4D). A significant difference was found in the ratios of the fluorescence intensity of dead/live cells between nisin in conjunction with 3D-AAs and 3L-AAs (*p*<0.05), and 3D-AA was found to be superior to 3L-AA (Fig. 5).

## Discussion

The use of D-amino acids is a recent strategy for combating biofilms. Some studies have indicated that D-amino acids can inhibit biofilm formation and disperse existing biofilms [5], [20], [21]. This study represents the first report of the effect of D-amino acids on *S. mutans* biofilm. Cys, Asp, and Glu at a concentration of 10 mM did not inhibit *S. mutans* growth but improved the antibacterial activity of nisin, and the three amino acids at a concentration of 40 mM prevented *S. mutans* biofilm formation. This finding indicates that the three amino acids may prevent *S. mutans* aggregation and may further help the antimicrobial peptide nisin exert its bactericidal activity. However, this enhancement was not observed with chlorhexidine and penicillin, and this lack of improvement may be related to their antibacterial mechanisms.

Nisin, a cationic and amphiphilic antibacterial peptide, can bind to the peptidoglycan precursor Lipid II serving as a docking molecule for subsequent pore formation, which results in a loss of the proton motive force and an efflux of small cytoplasmic compounds [22], [23], [24]. In previous studies, nisin showed certain antibacterial activities against nine cariogenic bacteria and was found to destroy the normal morphology of *S. mutans* [16], [17]. An organism generally has a restorable or self-repair function when suffering from nonlethal damage. Bacteria attacked by nisin may be able to self-repair the pores. D-amino acids are a key component of the cell wall peptidoglycan, which is essential for the maintenance of a high internal osmotic pressure inside the cell [25], [26]. The main structural features of peptidoglycan are linear glycan strands, which are composed of repeating disaccharide residues and are cross-linked via peptide side chains. The cross-linkage of the glycan strands commonly occurs between the distal carboxyl group of D-Ala at position 4 of a donor peptide and the free amino group of the diamino acid at position 3 of an acceptor peptide [25]. Thus, during the synthesis of peptidoglycan, the incorporation of some exogenous D-amino acids into the peptide side chains of the peptidoglycan in place of the terminal D-Ala may directly influence the cross-linking of the glycan strands and accordingly affect the strength and flexibility of peptidoglycan [27]. Thus, the restoration of the pore formed as a result of nisin treatment may be prevented through the addition of exogenous D-amino acids. Furthermore, the pore formed as a result of nisin treatment may facilitate the penetration of the exogenous D-amino acids into cells. D-amino acids are not normal metabolites in bacteria cells, and the penetration of exogenous D-amino acids may affect the normal metabolism of cells. Therefore, their occurrences may improve the antibacterial activity of nisin. In this study, we speculated that Cys, Asp, and Glu may affect bacterial plasticity and construction by disturbing the cross-linking of the glycan strands and thereby improve the antibacterial activity of nisin. This conjecture requires further study to be confirmed. The antibacterial mechanism of penicillin includes the binding of the penicillin-binding protein that catalyzes the last step of peptidoglycan biosynthesis [28]. The antibacterial action of CHX is through the interaction of the positively-charged molecule and the negatively-charged sites on the cell walls, thereby destabilizing the cell wall and interfering with the cell’s osmosis [29], [30]. The two antibacterial mechanisms appear to have no relevance to the cross-linkage of the glycan strands of peptidoglycan.

The combination of Cys, Asp, and Glu exerted a stronger antibiofilm activity than the components alone, which is similar to the synergetic effect of D-Leu, D-Met, D-Tyr, and D-Trp on the disassembly of *Bacillus subtilis* biofilms shown by Kolodkin-Gal *et al*. [5]. However, in our study, 3L-AAs prevented biofilm formation, and 3D-AAs did not disrupt existing biofilms. This finding indicates that the detachment of *S. mutans* biofilms by 3D- or 3L-AAs may not be more significant than the mixture of four D-amino acids used by Kolodkin-Gal *et al*. *S. mutans* biofilms contain a high amount of exopolysaccharide glucan, which helps cells tightly aggregate together and avoid detachment. A dispersant that can disrupt exopolysaccharide may be better able to disperse existing biofilms of *S. mutans*. Furthermore, 40 mM 3D-AAs and 3L-AAs at their original pH or at pH 7 can inhibit *S. mutans* growth, whereas the D-amino acids did not inhibit *Bacillus subtilis* growth in the study conducted by Kolodkin-Gal *et al.* The results indicate that 3D-AAs and 3L-AAs affect *S. mutans* biofilm formation likely through the inhibition of the bacteria.

Previous studies have indicated that nisin may be considered a potential anticariogenic compound. The present study showed that Cys, Asp, and Glu not only improve the antibacterial activity of nisin against *S. mutans* but also prevent its biofilm formation at the initial stage. The combination of these free amino acids with nisin may provide a strategy for the inhibition of the cariogenic bacteria *S. mutans* and biofilms. At present, the effect of the free amino acids on *S. mutans* biofilm is only an *in vitro* study, and the cariogenic bacteria in the dental plaque biofilms found in the oral cavity are very complex, and dental caries often involve multiple microorganisms. Further studies are in progress to evaluate the possible effect of these free amino acids and nisin on other cariogenic bacteria and biofilms. Furthermore, though amino acids belong to the vital nutrition of human being, the safety of the free amino acids at the higher concentration needs to further be studied in the topical application of oral cavity. Once the effect of the free amino acids on cariogenic bacteria and dental plaque biofilms is better understood, the combination of these amino acids with nisin may become a viable option for the prevention of dental caries.
